# Breast DCE-MRI morphological features and semi-quantitative parameters associated with upgrade to invasive carcinoma in biopsyproven DCIS

**DOI:** 10.2478/raon-2026-0043

**Published:** 2026-09-07

**Authors:** Vladimir Urban, Zorica Milosevic, Zoran Bukumiric, Katarina Obradovic, Nina Adzic, Dragana Pavlovic-Stankovic, Ivana Petkovic, Ivana Sovic, Irena Jovanic, Igor Spurnic

**Affiliations:** 1 Department of Diagnostic Radiology, Institute for Oncology and Radiology of Serbia, Belgrade, Serbia; 2 School of Medicine, University of Belgrade, Belgrade, Serbia; 3 Institute for Medical Statistics and Informatics, Belgrade, Serbia; 4 Department of Pathology, Institute for Oncology and Radiology of Serbia, Belgrade, Serbia; 5 Department of Surgery, Institute for Oncology and Radiology of Serbia, Belgrade, Serbia

**Keywords:** DCIS, breast biopsy, breast MRI, semi-quantitative, upgrade, invasive carcinoma

## Abstract

**Background:**

The aim of this study was to identify imaging predictors of upgrade to invasive carcinoma (IC) in patients with biopsy-proven ductal carcinoma *in situ* (DCIS) based on morphological features and semi-quantitative parameters derived from dynamic contrast-enhanced breast magnetic resonance imaging (DCE-MRI).

**Patients and methods:**

Ninety consecutive patients with biopsy-proven DCIS who had positive findings on standard full-protocol breast DCE-MRI and subsequently underwent surgical excision with final postoperative histopathological diagnosis were included. Patients with final postoperative IC were compared with those without IC. Morphological features (lesion type, size, multifocality/multicentricity, T2-weighted (T2W) and turbo inversion recovery magnitude [TIRM] characteristics) and semi-quantitative parameters (wash-in, peak enhancement, delayed phase of the timeintensity curve [TIC], time to peak [TTP], and positive enhancement integral [PEI]) were analyzed.

**Results:**

Fifty-five patients (61.1%) had no IC, while 35 patients (38.9%) had IC on final histopathology. Patients with IC more frequently demonstrated non-mass enhancement (NME) combined with a mass, larger lesion size, multifocal or multicentric disease, T2W/TIRM hyperintensity, fast wash-in, higher peak enhancement, plateau and washout TIC patterns, higher PEI, and shorter TTP. Multivariate analysis identified NME combined with a mass, delayed-phase TIC characteristics, and TIRM hyperintensity as independent predictors of IC.

**Conclusions:**

Our study confirmed that prediction models incorporating breast DCE-MRI morphological features and semi-quantitative parameters can stratify patients with biopsy-proven DCIS by their probability of upgrade to IC. TIRM hyperintensity emerged as one of three independent predictors of IC and, to the best of our knowledge, has not been previously reported in this context.

## Introduction

The World Health Organization defines ductal carcinoma *in situ* (DCIS) as a neoplastic proliferation of epithelial cells confined to the mammary ductallobular system, characterized by subtle to marked cytological atypia and an inherent, though not necessarily obligate, tendency to progress to invasive cancer.^1,2^ When the integrity of the basement membrane is disrupted and the tumor extends beyond the ductal-lobular system into adjacent tissue, it is classified as invasive carcinoma (IC). Because DCIS is considered a non-obligate precursor of IC, timely detection and treatment may prevent progression to invasive disease. Currently, in the era of widespread screening mammography, most newly diagnosed cases of DCIS are asymptomatic (80–85%) and are detected incidentally.^2^ Mammography remains the standard modality for DCIS detection due to its high sensitivity for suspicious microcalcifications, which are present in about 75% of DCIS cases.^3^

DCIS can be unifocal or involve multiple segments of one or more ductal systems, with intervening normal breast tissue.^4^ In addition, DCIS may coexist with IC, reflecting the heterogeneous nature of malignant lesions. Consequently, percutaneous biopsy may underestimate the presence of invasive disease due to sampling limitations and intralesional heterogeneity. In clinical practice, the identification of IC on final postoperative histopathology following a percutaneous biopsy diagnosis of DCIS is regarded as an “upgrade”. Preoperative identification of a possible concomitant IC in the cases of percutaneously diagnosed DCIS is important, as it guides further patient management, particularly the need for sentinel lymph node biopsy (SLNB).

In this context, dynamic contrast-enhanced breast magnetic resonance imaging (DCE-MRI) may provide additional information. Following intravenous administration of a low-molecular-weight gadolinium-based contrast agent to assess tumor angiogenesis, DCE-MRI detects areas of pathological enhancement that cannot be visualized on mammography or ultrasound.^4^ By combining morphological features and dynamic enhancement parameters, DCE-MRI offers the greatest potential among breast imaging modalities for excluding IC.^5^ According to the literature, the sensitivity of DCE-MRI for detecting DCIS ranges from 88% to 92%, while sensitivity for detecting IC ranges from 91% to 95%.^5-9^ However, considerable controversy remains regarding the usefulness of breast DCE-MRI in patients with preoperatively biopsy-proven DCIS, as published results are highly variable and sometimes conflicting.^5,10-22^

In this article, we focused on the morphological features of preoperatively biopsy-proven DCIS and the time-intensity curve (TIC) of its most enhancing part as a semi-quantitative parameter, both derived from a standard full breast DCE-MRI protocol and interpreted in accordance with the ACR BI-RADS lexicon.^23^ We also analyzed two additional semi-quantitative parameters derived from TIC – the positive enhancement integral (PEI) and time to peak (TTP). These parameters are readily available in vendor-provided software packages. PEI and TTP can be expressed both numerically and graphically (as color maps), reflecting different aspects of tissue perfusion. Although they are not part of the BI-RADS framework, PEI and TTP are suitable for research purposes and may also be applicable in routine clinical practice, as they can assist radiologists in identifying areas of suspicious enhancement.^24^

All semi-quantitative parameters are based on the premise that malignant lesions tend to take up and release the contrast agent earlier and faster than benign lesions. TIC represents the change in signal intensity (%) over time (s). It is generated for each voxel or a selected region of interest (ROI) and is derived from multiple serial images that are obtained after contrast agent injection. TIC comprises an initial phase (wash-in), representing the initial rate of contrast enhancement (slow, medium, or fast), and a delayed phase, during which signal intensity (SI) may continue to rise (type 1 – persistent), reach a steady level (type 2 – plateau), or decrease (type 3 – washout).^23^ PEI is defined as the integral of the area under the TIC and above the baseline and reflects the concentration of contrast agent within the selected ROI at a given time point.^22,25,26^ TTP is the time (s) required for SI to reach its peak value after contrast agent injection; therefore, more avid contrast uptake is associated with shorter TTP values.^18^ For both parameters, color maps can be automatically generated for each slice of a breast DCE-MRI examination, providing a clear overview of areas with suspicious enhancement.

On DCE-MRI, DCIS most commonly appears as areas of clumped non-mass enhancement (NME), usually with a linear or segmental distribution, similar to microcalcifications on mammography.^27^ Less common presentations include focal or regional NME, foci, masses, or architectural distortion. The TIC of contrast enhancement in DCIS typically demonstrates fast wash-in during the initial phase; however, in the delayed phase, all three curve types may be observed, most commonly plateau, but also persistent and washout patterns.^4,28^ Nevertheless, because DCIS lacks intrinsic vascularity and enhances predominantly by diffusion of gadolinium contrast agent, its enhancement pattern differs from that of IC, with slower wash-in and rare washout.^29^ Consequently, DCIS is associated with longer TTP values and lower PEI values compared with IC.^22,30^

The aim of this study was to: (1) compare morphological features and semi-quantitative breast DCE-MRI parameters in patients with preoperatively biopsy-proven DCIS, stratified by final postoperative histopathology (without IC *vs*. with IC); (2) identify independent imaging predictors of IC and to develop a predictive model for detecting coexistent IC.

## Patients and methods

### Patients

This retrospective study included patients with DCIS diagnosed by percutaneous breast biopsy between January 2013 and December 2022 at the Institute for Oncology and Radiology of Serbia (Belgrade, Serbia). All patients provided written informed consent prior to the DCE-MRI examination. The study was approved by the institutional Ethics Committee (approval No. 3926-01).

The inclusion criteria were: (1) availability of a preoperative standard full-protocol breast DCE-MRI demonstrating an enhancing lesion; (2) imaging-guided percutaneous biopsy performed either after DCE-MRI or at least 14 days before DCE-MRI; (3) biopsy performed using one of the following techniques: ultrasound-guided 14-gauge core-needle biopsy (CNB), stereotactic or digital breast tomosynthesis–guided 9-gauge vacuum-assisted biopsy (SVAB or DBT-VAB), or magnetic resonance–guided 9-gauge vacuum-assisted biopsy (MR-VAB); (4) a mean of five cores for CNB and twelve for vacuum-assisted biopsy techniques^14^; (5) subsequent surgical excision of the lesion; (6) availability of a final postoperative histopathological diagnosis; and (7) histopathological evaluation performed by specialized oncologic pathologists at the same institution.

The exclusion criteria were: (1) inability to tolerate the DCE-MRI examination; and (2) severe image artifacts that reduced the sensitivity and specificity of diagnostic interpretation.

According to these criteria, 90 consecutive patients with DCIS diagnosed by percutaneous breast biopsy were included. Based on the final postoperative histopathological diagnosis, patients were divided into two groups: those without IC and those with IC, the latter being defined as upgrade to IC. The group without IC comprised patients with pure DCIS on final histopathology as well as patients without residual DCIS or IC, as confirmed by the postoperative multidisciplinary board.

### Standard full breast DCE-MRI protocol and postprocessing

All breast DCE-MRI examinations were performed using 1.5 T Siemens scanners (Siemens Medical Solutions, Erlangen, Germany): a Magnetom Avanto system until February 2018 and a Magnetom Avanto Fit system from April 2018 onward, both equipped with a dedicated bilateral breast coil. Premenopausal patients were scanned during the second week of their menstrual cycle to minimize false-positive findings. Images were acquired in the prone position using a 2-mm slice thickness according to the following protocol: axial turbo-spin-echo (TSE) T2-weighted (T2W) without fat suppression (FS) (echo time 70 ms, repetition time 5900 ms, flip angle 180°, field of view 340 × 340 mm, image matrix 384 × 319), turbo inversion recovery magnitude (TIRM, echo time 60 ms, repetition time 7690 ms, inversion time 150 ms, flip angle 150°, field of view 340 × 340 mm, image matrix 320 × 256), axial TSE T1-weighted (T1W) without FS (echo time 12 ms, repetition time 910 ms, flip angle 90°, field of view 340 × 340 mm, image matrix 320 × 234), and dynamic three-dimensional fast low-angle shot (3D FLASH) sequences without FS (echo time 4.8 ms, repetition time 9.1 ms, flip angle 25°, field of view 340 × 340 mm, image matrix 576 × 564), comprising one pre-contrast acquisition followed by five successive acquisitions every 1 min 23 s after intravenous contrast agent administration. The gadolinium-based contrast agents used were gadopentetate dimeglumine (Magnevist, Bayer Schering Pharma, Berlin, Germany) during the initial study period (until June 2014) and gadobutrol (Gadovist, Bayer Schering Pharma, Berlin, Germany) thereafter. This transition from a linear to a macrocyclic gadolinium-based contrast agent reflected evolving safety recommendations regarding gadolinium retention.^31^ Contrast agents were administered as a bolus injection at a dose of 0.1 mmol/kg using an automatic injector (Mississippi, Ulrich Medical, Ulm, Germany) at a rate of 2 ml/s, followed by a 20 ml saline flush.

The indications for breast DCE-MRI were determined by the institutional preoperative multidisciplinary board in accordance with national guidelines for breast cancer diagnosis and treatment.^32^

### Image analysis

Images were analyzed using Syngo or Syngo Via software (Siemens Medical Solutions, USA). Postprocessing was performed to generate subtraction images, multiplanar reconstructions, TICs, and parametric colour maps, including wash-in, TTP, and PEI maps. Additionally, PEI values were normalized by calculating a lesion-to-normal parenchyma (L/NP) ratio, defined as the ratio of lesion PEI to the PEI measured at the corresponding location in the contralateral normal breast parenchyma. The normalization was applied to minimize inter-patient and inter-scan variability related to differences in MRI scanners and contrast agents.^22^ All DCE-MRI examinations were independently reviewed by two radiologists with 8 years (V. U.) and 12 years (D. P. S.) of clinical experience in breast DCE-MRI, who were blinded to all patient-related information, including histopathological diagnosis. Discrepancies were resolved by consensus, and the final consensus assessment was used for statistical analysis.

The following DCE-MRI data were evaluated: morphological features (lesion type – mass, NME or both; lesion size; multifocality/multicentricity; and T2W and TIRM signal characteristics) and semi-quantitative parameters, including TIC characteristics (wash-in, peak enhancement, and delayed phase), TTP, PEI, and the PEI L/NP ratio. For wash-in analysis, enhancement thresholds were defined as ≥ 50% for medium enhancement and ≥ 100% for fast enhancement.^23^ A freehand ROI (14.37 ± 9.68 mm^2^) was placed over the fastest enhancing part of the lesion, as assessed on the wash-in map – a parametric color map reflecting enhancement behavior across the dynamic postcontrast acquisition, with higher signal intensity corresponding to faster wash-in – and was then copied onto the PEI and TTP maps to calculate the corresponding values. For the purpose of semi-quantitative analysis, in cases of lesion multiplicity, the dominant lesion was defined as the lesion with the highest signal intensity on the wash-in map, corresponding to the fastest wash-in. In cases of discrepancies in ROI placement and/or dominant lesion selection, consensus was used to determine the final ROI and/or dominant lesion for statistical analysis.

### Statistical analysis

Statistical analysis was performed using IBM SPSS Statistics version 21 (IBM Corp., Armonk, NY, USA) and R software version 4.5.0 (R Foundation for Statistical Computing, Vienna, Austria). Normally distributed variables were expressed as mean ± standard deviation, whereas non-normally distributed variables were presented as median and interquartile range (IQR). Categorical variables were described using frequencies and percentages. Depending on variable type, between-group differences were assessed using appropriate parametric (Student’s t-test) or non-parametric tests (χ^2^, Fisher’s exact test, or Mann-Whitney U test). The least absolute shrinkage and selection operator (LASSO) algorithm was used for variable selection prior to multivariate logistic regression analysis. Model fit was evaluated using the likelihood ratio test, Hosmer-Lemeshow goodness-of-fit test, and Nagelkerke’s pseudo-R^2^. Diagnostic performance was assessed using receiver operating characteristic (ROC) analysis and area under the curve (AUC). The optimal cut-off value was determined using the Youden index, and sensitivity and specificity were calculated accordingly. P-values < 0.05 were considered statistically significant.

## Results

Based on final postoperative histopathology, among patients without IC, pure DCIS was confirmed in 49 of 90 patients (54.4%), while no residual DCIS was identified in 6 patients (6.7%). IC was found in 35 of 90 patients (38.9%), including 7 cases (7.8%) of microinvasive carcinoma. Regarding histological types, intracystic papillary carcinoma was present in 2 cases (2.2%), while tubular, tubulo-lobular, mucinous, mixed ductal and mucinous, lobular and metaplastic carcinoma were each identified in 1 case (1.1%). The remaining invasive carcinomas were of no special type (NST). These 35 patients (38.9%) therefore represented an upgrade from the initial percutaneous biopsy diagnosis of DCIS.

**FIGURE 1.**
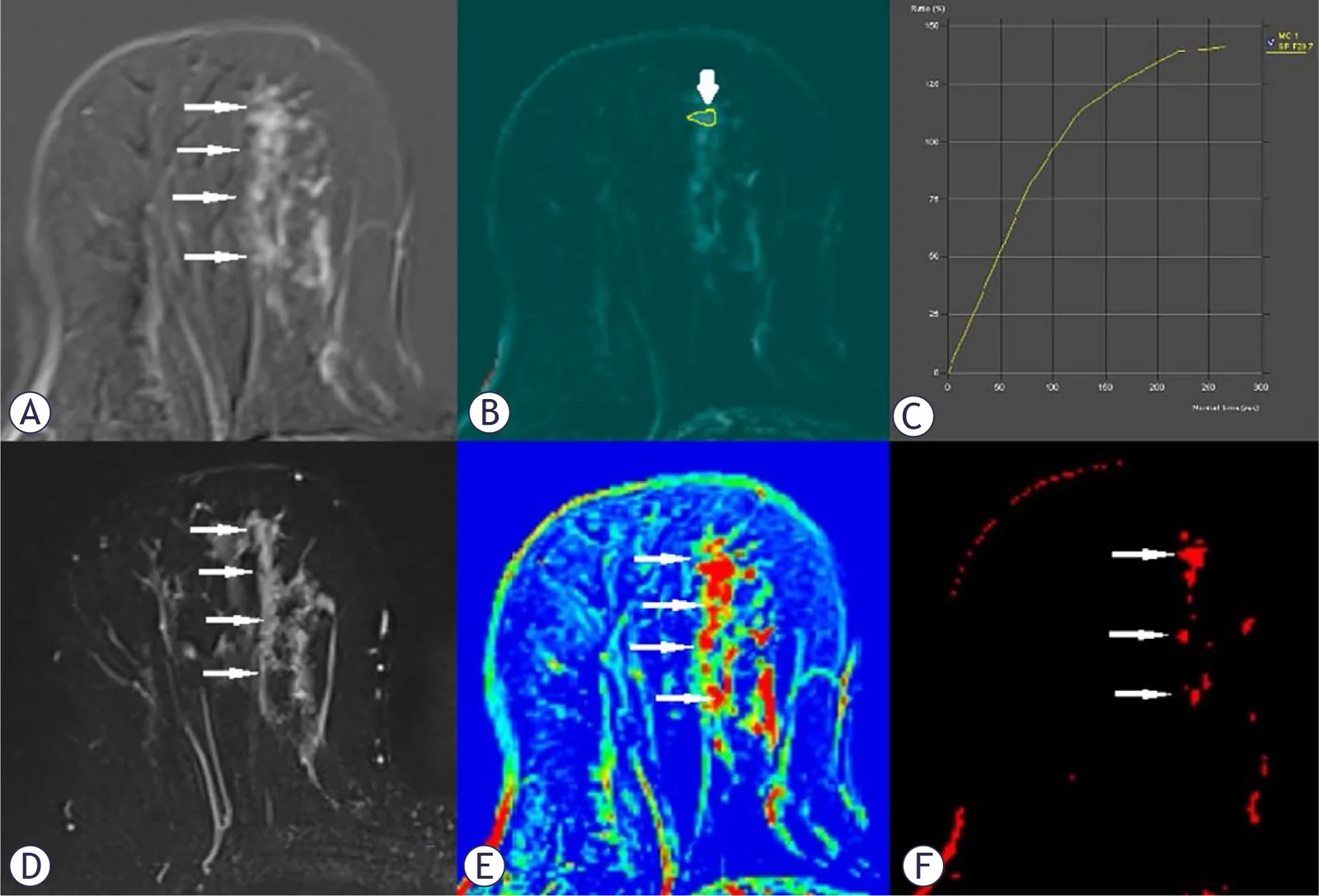
A 69-year-old woman with biopsy-proven ductal carcinoma *in situ* (DCIS) presenting as segmental clumped nonmass enhancement (NME) on breast dynamic contrast-enhanced magnetic resonance imaging (DCE-MRI) (thin arrows). The upper row shows the dynamic contrast-enhanced study: subtracted postcontrast image demonstrating the extent and distribution of NME **(A)**, wash-in parametric map **(B)**, and time-intensity curve (TIC) analysis **(C)**. On the wash-in map, the freehand region of interest (ROI) was placed over the fastest enhancing part of the lesion, corresponding to the area with the highest signal intensity (thick arrow). TIC analysis demonstrated medium initial enhancement and a persistent delayed phase, corresponding to type 1 kinetics. The lower row shows the corresponding turbo inversion recovery magnitude (TIRM) image with no hyperintense areas **(D)**, positive enhancement integral (PEI) map **(E)**, and time to peak (TTP) map **(F)**, illustrating the semi-quantitative parameters used for lesion assessment. Final postoperative histopathology confirmed pure DCIS without invasive carcinoma (IC).

**FIGURE 2.**
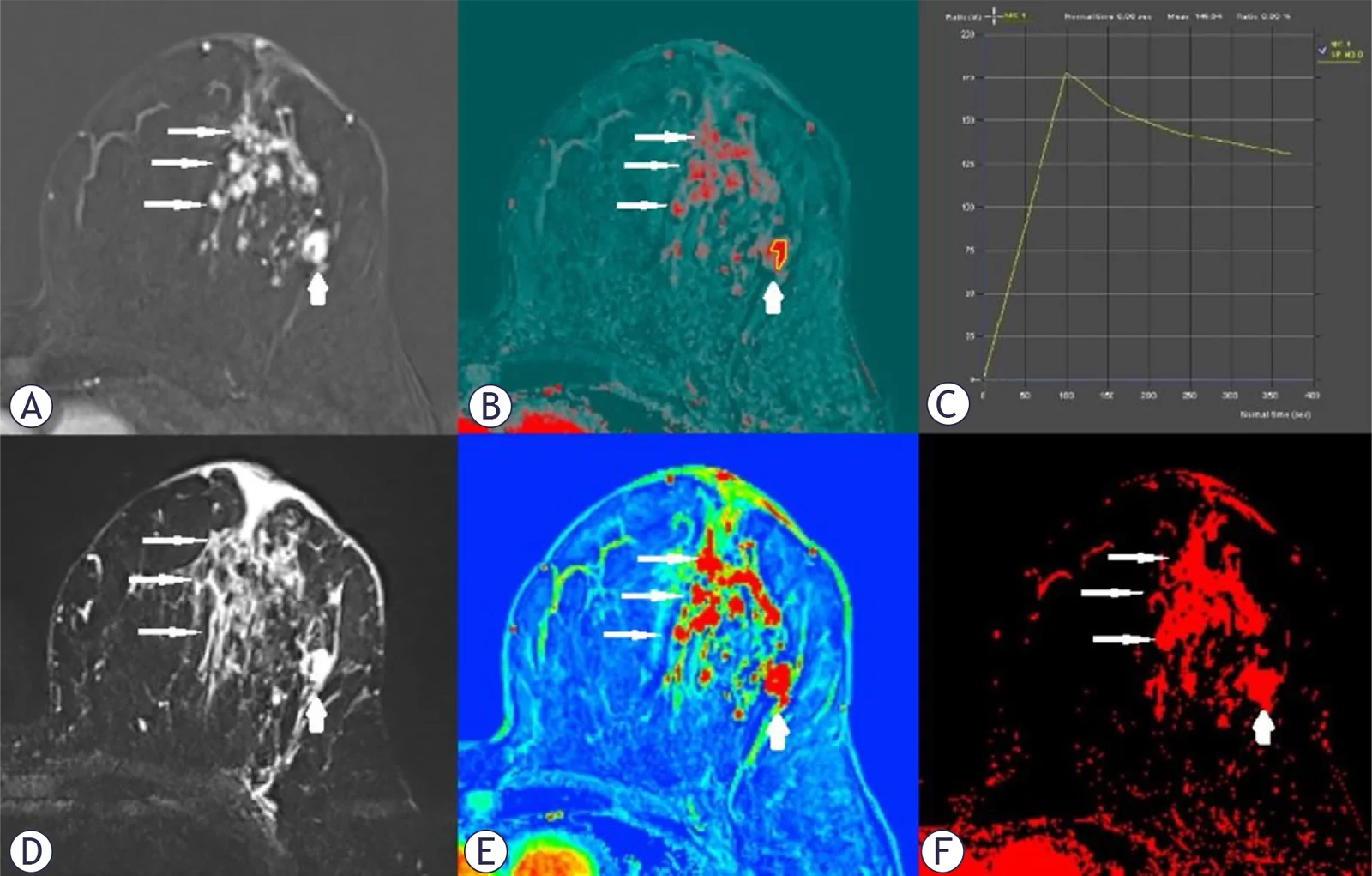
A 56-year-old woman with biopsy-proven ductal carcinoma *in situ* (DCIS) presenting as segmental non-mass enhancement (NME) (thin arrows) with an associated irregular mass in the inner quadrants (thick arrow) on breast dynamic contrast-enhanced magnetic resonance imaging (DCE-MRI). The upper row shows the dynamic contrast-enhanced study: subtracted postcontrast image demonstrating the extent of NME and the associated irregular mass **(A)**, wash-in parametric map **(B)**, and time-intensity curve (TIC) analysis **(C)**. On the wash-in map, the freehand region of interest (ROI) was placed over the irregular mass, which represented the fastest enhancing part of the lesion and corresponded to the area with the highest signal intensity (thick arrow). TIC analysis demonstrated fast initial enhancement and washout delayed-phase kinetics, corresponding to type 3 kinetics. The lower row shows the corresponding turbo inversion recovery magnitude (TIRM) image, where the lesion appears conspicuously hyperintense **(D)**, positive enhancement integral (PEI) map **(E)**, and time to peak (TTP) map **(F)**, illustrating the semi-quantitative parameters used for lesion assessment. Final postoperative histopathology revealed DCIS with an associated invasive carcinoma (IC).

Patient characteristics, as well as morphological and semi-quantitative breast DCE-MRI findings for these two groups and their corresponding statistical comparisons, are presented in Table 1 and Figures 1 and 2.

**TABLE 1.** Comparison of findings between patients with and without invasive carcinoma

	Without IC (n = 55)	With IC (n = 35)	p-value
Age	55.29 ± 11.13	51.91 ± 9.42	0.141
Type of biopsy			
CNB	19 (34.5%)	17 (48.6%)	0.256
SVAB/DBT-VAB	34 (61.8%)	18 (51.4%)	
MR-VAB	2 (3.6%)	0	
DCE-MRI lesion type			
NME	45 (81.8%)	14 (40%)	< 0.001*
Mass	6 (10.9%)	6 (17.1%)	
NME + mass	4 (7.3%)	15 (42.9%)	
Lesion size (mm)	25 (52)	48 (46)	0.015*
Multifocality/multicentricity			
No	37 (67.3%)	10 (28.6%)	< 0.001*
Yes	18 (32.7%)	25 (71.4%)	
Wash-in			
Slow	13 (23.6%)	3 (8.6%)	< 0.001*
Medium	18 (32.7%)	2 (5.7%)	
Fast	24 (43.6%)	30 (85.7%	
Peak enhancement (%)	253.49 ± 168.06	343.14 ± 195.26	0,023*
Delayed phase			
Persistent	34 (61.8%)	5 (14.3%)	< 0.001*
Plateau	14 (25.5%)	16 (45.7%)	
Washout	7 (12.7%)	14 (40%)	
T2W TSE			
Hypointense	19 (34.5%)	8 (22.9%)	0.023*
Isointense	32 (58.2%)	17 (48.6%)	
Hyperintense	4 (7.3%)	10 (28.6%)	
TIRM			
Hypointense	14 (25.5%)	6 (17.1%)	0.003*
Isointense	23 (41.8%)	5 (14.3%)	
Hyperintense	18 (32.7%)	24 (68.6%)	
PEI	988 (733)	1391 (1307)	0.009*
PEI L/NP	10.83 (7.61)	11 (11.92)	0.493
TTP	470 (343)	359 (182)	0.003*

CNB = core-needle biopsy; DBT-VAB = digital breast tomosynthesis-guided vacuum-assisted biopsy; DCE-MRI = dynamic contrast-enhanced magnetic resonance imaging; IC = invasive carcinoma; L/NP = lesion-to-normal parenchyma; MR-VAB = magnetic resonance-guided vacuum-assisted biopsy; NME = non-mass enhancement; PEI = positive enhancement integral; SVAB = stereotactic vacuum-assisted biopsy; T2W TSE = T2-weighted turbo-spin-echo; TIRM = turbo inversion recovery magnitude; TTP = time to peak.

Normally distributed numeric variables are presented as mean ± standard deviation, non-normally distributed variables as median (interquartile range), and categorical variables as frequencies (percentages). Statistically significant differences are marked with an asterisk (*).

Compared with patients without IC, patients with IC showed a significantly higher prevalence of the following morphological DCE-MRI features: NME combined with a mass (p < 0.001), larger lesion size (p = 0.015), multifocal or multicentric disease (p < 0.001), T2W hyperintensity (p = 0.023), and TIRM hyperintensity (p = 0.003).

Regarding semi-quantitative parameters, patients with IC more frequently exhibited plateau and washout TIC patterns (p < 0.001), fast wash-in (p < 0.001), higher peak enhancement (p = 0.023), higher PEI values (p = 0.009), and shorter TTP (p = 0.003).

No significant differences between the two groups were observed with respect to age (p = 0.141), biopsy type (p = 0.256), or the PEI L/NP ratio (p = 0.493).

After application of the LASSO variable selection algorithm to all tested variables, four predictors were selected for inclusion in multivariate logistic regression analysis: lesion type (NME, mass, or NME combined with a mass), multifocality/multicentricity, delayed-phase TIC characteristics, and TIRM characteristics. These variables were entered into the multivariate logistic regression model, which identified NME combined with a mass, delayed-phase TIC characteristics, and TIRM hyperintensity as independent predictors of IC (Table 2).

**TABLE 2.** Multivariate regression analysis with independent predictors of invasive carcinoma

Variable	OR	95% CI	p-value
NME + mass	4.494	1.078–18.742	0.039*
Multifocality/multicentricity	2.407	0.716–8.096	0.156
Delayed phase	3.915	1.665–9.205	0.002*
TIRM-hyperintensity	6.754	1.448–31.506	0.015*

CI = confidence interval; NME = non-mass enhancement; OR = odds ratio; TIRM = turbo inversion recovery magnitude.

Statistically significant independent predictors are marked with an asterisk (*).

The overall model was statistically significant (p < 0.001), with no evidence of relevant multicollinearity among predictors. Nagelkerke’s R^2^ indicated that approximately 50.5% of the variance in invasive disease status was explained by the model.

**FIGURE 3.**
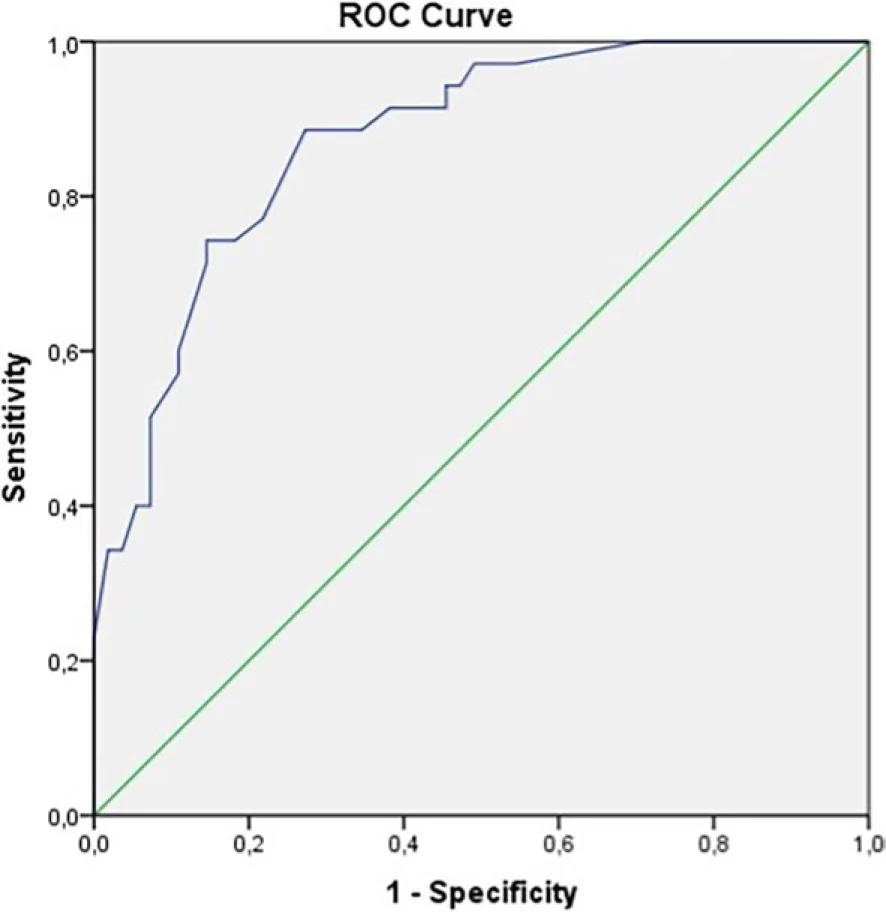
Receiver operating characteristic (ROC) curve for the multivariate regression model (area under the curve [AUC] = 0.872).

ROC analysis yielded an AUC of 0.872 (Figure 3). The optimal probability cut-off was 0.31, corresponding to a sensitivity of 88.6% and specificity of 72.7%.

## Discussion

The results of our study suggest that morphological and dynamic features described in the BI-RADS atlas, as well as additional semi-quantitative parameters derived from breast DCE-MRI, may contribute to the preoperative prediction of upgrade to IC in patients with biopsy-proven DCIS. The comparison between patients with and without IC was based on postoperative histopathology as the reference standard for defining upgrade, reflecting the clinical aim of identifying patients at increased risk of upgrade before surgery. These findings are discussed in relation to previous studies addressing the same clinical question.

In our study, 38.9% of patients with biopsyproven DCIS were upgraded to IC after surgery, which falls within the range reported in previous studies. Postoperative upgrade rates of 25–43% have been described, including 25% reported by Wisner *et al*., 40.7% by Takada *et al*., 36.4% underestimation of invasive disease on ultrasound-guided CNB reported by Park M *et al*., 38% by Miceli *et al*., and 33% by Dillon *et al*., who additionally reported microinvasive carcinoma in 14%, and suspected microinvasion in 7.5% of cases.^5,10-13^ Yoon *et al*. reported pure DCIS in 54.4% of patients, microinvasive carcinoma in 24.3%, and IC in 21.4%, while Lee CW *et al*. reported IC in 43% of patients and noted a broad upgrade range of 3.5–56% in the literature.^14,15^

In our cohort, 6 patients (6.7%) had no residual DCIS on final histopathology, reflecting complete removal of the lesion at biopsy, as confirmed by the multidisciplinary board. Similar findings have been reported previously, with Wisner *et al*. observing no residual DCIS or invasion in 8% of patients and Lee KH *et al*. reporting this outcome in 2% of cases.^5,16^

The upgrade rate was higher among patients who underwent ultrasound-guided CNB (47.2%) compared with those who underwent vacuum-assisted biopsy (33.3%), although this difference did not reach statistical significance (p = 0.185). In contrast, other studies have demonstrated statistically significant differences. Takada *et al*. reported a significantly higher postoperative upgrade rate in CNB compared with VAB, with biopsy method identified as an independent predictor of invasion on multivariate analysis.^10^ Dillon *et al*. similarly reported a higher upgrade rate after ultrasound-guided CNB than after stereotactic techniques, with a statistically significant difference, while Brennan *et al*., in a meta-analysis, found higher underestimation rates with 14-gauge automated biopsy devices than with 11-gauge VAB.^13,17^ This may be partly explained by differences in lesion presentation and sampling volume. Ultrasound-guided CNB is more often performed for larger lesions or palpable masses, which are more likely to be associated with IC, whereas SVAB/DBT-VAB and MR-VAB are frequently performed for microcalcifications or NME, which are more commonly associated with pure DCIS. In addition, the smaller tissue volume obtained with CNB may increase the risk of sampling error and missing an invasive component.^17^

In our study, patients with IC (mean age 51.91 ± 9.42) were younger than patients without IC (mean age 55.29 ± 11.13), but the difference was not statistically significant (p = 0.141). Similar findings of younger age in patients with IC than in patients without IC, but without a statistically significant difference, were reported by Ko *et al*. and Park M *et al*.^11,18^

In contrast, lesion size was significantly larger in patients with IC (median 48 mm *vs*. 25 mm, p = 0.015). This is consistent with several previous studies and may be explained by the greater likelihood of unsampled invasive foci within larger areas of DCIS.^13,18,19^ Dillon *et al*. reported that lesions ≥ 5 cm on postoperative histopathology were more likely to harbor invasive disease.^13^ Ko *et al*. found that the longest tumor diameter was significantly larger in IC than in pure DCIS and identified DCIS size as an independent predictor of IC on multivariate analysis.^18^ Takada *et al*. reported a significantly higher probability of IC when tumor diameter exceeded 20 mm, Goto *et al*. observed larger lesions in patients with IC, while Park AY *et al*. found a significantly higher upgrade rate in lesions ≥ 30 mm.^10,19,20^ In contrast, Lee CW *et al*. found no significant difference in lesion size measured by DCE-MRI, although tumor volume was significantly greater in patients with IC, while Park M *et al*. reported larger lesions in patients without underestimation, without statistical significance.^11,15^

Multifocal and multicentric lesions were significantly more frequent in patients with IC than in those without IC (71.4% *vs*. 32.7%, p < 0.001); however, this parameter was not an independent predictor in the multivariate regression model (OR = 2.407, 95% CI = 0.716–8.096, p = 0.156). In contrast, Lee CW *et al*. found no significant difference in the frequency of multifocal/multicentric disease between IC and pure DCIS.^15^

In our study, lesion type on DCE-MRI differed significantly between patients with and without IC (p < 0.001), and the combined presence of a mass and NME emerged as an independent predictor of IC on multivariate analysis (OR = 4.494, 95% CI = 1.078–18.742, p = 0.039). Wisner *et al*. showed that the presence of a mass was strongly associated with invasion for both independent readers, whereas NME was negatively associated with invasion for only one reader.^5^ Lee CW *et al*. also found a significantly higher rate of mass-like lesions in patients with IC than in those with pure DCIS.^15^ In contrast, Yoon *et al*. reported that both masses and NME were independent predictors of histological upgrade, with NME being the stronger predictor, while Goto *et al*. found no statistically significant difference in lesion type between DCIS and IC.^14,20^

In our study, both T2W and TIRM hyperintensity were more prevalent in patients with IC, reaching statistical significance (p = 0.023 and p = 0.003, respectively). TIRM hyperintensity was also identified as an independent predictor on multivariate analysis (OR = 6.754, 95% CI = 1.448–31.506, p = 0.015). A possible explanation is that invasive lesions tend to have higher tissue water content due to necrosis, edema, and/or inflammatory changes, and TIRM is more sensitive to increased water content than conventional T2W TSE imaging. Data from previous studies are conflicting. Goto *et al*. reported that high signal intensity (SI) on fat-suppressed T2W TSE (similar to TIRM) was significantly more common in patients with IC.^20^ In contrast, Park AY *et al*. reported that lower signal intensity on fat-saturated T2W imaging was associated with a significantly higher underestimation rate.^19^ Wisner *et al*. and Lee CW *et al*. found no significant correlation between T2W signal characteristics and invasion.^5,15^

To the best of our knowledge, TIRM hyperintensity has not previously been specifically evaluated as an independent predictor of upgrade to IC in patients with biopsy-proven DCIS. Although conventional DCE-MRI descriptors, including lesion morphology and enhancement kinetics, have been associated with invasive disease or upgrade in previous studies, TIRM provides information derived from a non-contrast, fluid-sensitive sequence and may therefore reflect tissue characteristics not fully captured by dynamic enhancement analysis. In the present study, TIRM hyperintensity remained an independent predictor in the multivariate regression model together with lesion type and delayed-phase TIC characteristics, suggesting that it may provide complementary information beyond established morphological and kinetic DCE-MRI descriptors. This finding may be clinically relevant because TIRM is routinely available as part of standard breast DCE-MRI protocols, requires no additional contrast administration or advanced postprocessing, and may contribute to preoperative risk stratification in patients with biopsy-proven DCIS. However, given the limited sample size and retrospective design of our study, this observation should be regarded as hypothesis-generating and requires validation in larger prospective cohorts.

Three aspects of TIC were analyzed in our study: wash-in, peak enhancement, and delayed phase kinetics. Fast wash-in, higher peak enhancement, and plateau or washout delayed-phase patterns were more frequent in patients with IC (p < 0.001, p = 0.023, and p < 0.001, respectively), whereas persistent enhancement was more common in patients without IC. Delayed-phase TIC characteristics were additionally identified as an independent predictor of IC on multivariate regression analysis (OR = 3.915, 95% CI = 1.665–9.205, p = 0.002). These findings are largely consistent with previous studies. Wisner *et al*. reported that fast wash-in and washout delayed-phase kinetics were positively associated with invasion, while persistent delayed enhancement showed a negative association; however, all three parameters reached statistical significance for only one of the two independent readers.^5^ Ko *et al*. found that a higher signal enhancement ratio (SER), reflecting faster wash-in and washout, was an independent predictor of IC with a cut-off value > 0.7, together with larger DCIS size.^18^ Deurloo *et al*. demonstrated that the absence of enhancement or a persistent TIC were the strongest predictors for excluding invasive disease on multivariate analysis.^21^ In contrast, Park M *et al*. and Lee CW *et al*. did not find statistically significant differences in peak or initial enhancement, although some trends toward stronger enhancement in upgraded lesions were observed.^11,15^

To date, PEI and the L/NP ratio for PEI have not been extensively evaluated as predictors of upgrade from biopsy-proven DCIS to IC. In our study, PEI values were significantly higher in patients with IC (median 1391 *vs*. 988; p = 0.009), whereas the L/NP ratio for PEI did not differ significantly between groups (p = 0.493). Nadrljanski *et al*. reported higher PEI values in invasive ductal carcinoma than in DCIS, as well as significant differences between lesions and contralateral normal breast parenchyma.^22^ However, their study was based on diagnoses obtained after biopsy rather than final postsurgical histopathology, which may partly explain differences between studies.

In our cohort, TTP values were significantly lower in patients with IC, reflecting faster enhancement (median 359 *vs*. 470; p = 0.003). Ko *et al*. evaluated TTP among other DCE-MRI parameters and observed shorter TTP values in patients with IC, although the difference was not statistically significant.^18^

It is worth noting that our study focused on semi-quantitative parameters that are part of widely available software packages and easily applicable in routine clinical practice. In contrast, there is growing interest in fully quantitative parameters, which require more demanding technical implementation and are currently used primarily in research settings.^33,34^

As discussed above, from a clinical standpoint, the ability to identify patients at higher risk of upgrade prior to surgery remains highly relevant, regardless of whether the invasive component was not sampled at biopsy or whether the imaging findings overlap with general imaging characteristics of invasive carcinoma. In this context, the identified DCE-MRI features may provide useful information for preoperative risk stratification and surgical planning. Literature data on the predictive value of individual DCE-MRI morphological features and semi-quantitative parameters for detecting IC in biopsy-proven DCIS are highly variable, and no single parameter allows sufficiently accurate prediction. However, when combined in a multivariate regression model, their predictive performance may improve. In our study, we developed a statistically significant model (p < 0.001) with a strong overall fit (Nagelkerke’s R^2^ = 50.5%) and an AUC of 0.872, indicating excellent discrimination between cases with and without IC. In this model, the presence of both NME and a mass, delayed-phase TIC characteristics, and TIRM hyperintensity emerged as independent predictors of upgrade to IC.

This study had several limitations. First, the sample size was relatively small, which limits the robustness and generalizability of the findings, particularly in the context of multivariable prediction modeling. Second, the study was retrospective, and potential selection bias cannot be excluded. Third, although all DCE-MRI examinations were independently reviewed by two radiologists and final assessments were established by consensus, interobserver variability was not formally analyzed. Fourth, clinical, mammographic, ultrasound, and detailed histopathological data were not analyzed, as the focus of this study was on multiparametric breast DCE-MRI with additional semi-quantitative parameters.

In conclusion, DCE-MRI-based prediction models incorporating morphological features and semi-quantitative parameters may help stratify patients according to the risk of upgrade at surgery. In our cohort, the combined presence of NME and a mass, delayed-phase TIC characteristics, and TIRM hyperintensity were independent predictors of upgrade to IC. Notably, TIRM hyperintensity has not, to the best of our knowledge, been specifically evaluated as an independent predictor of upgrade to IC in this context. Although PEI and TTP differed significantly between patients with and without IC, their independent predictive value was limited. Given the paucity of existing literature data on these parameters, further prospective cohort studies are warranted to validate their role in breast DCE-MRI assessment and to evaluate whether incorporation of multimodal clinical, mammographic, ultrasound, and histopathological parameters could further improve preoperative prediction of upgrade.
